# Supplementary material for: Simultaneous rift-scale inflation of a deep crustal sill network in Afar, East Africa
Source: Nat Commun. 2024 May 20;15:4287. doi: 10.1038/s41467-024-47136-4 (PMC11893136; doi:10.1038/s41467-024-47136-4)
Supplement: Supplementary file 1 — Supplementary Information [file 41467_2024_47136_MOESM1_ESM.pdf]

Supplementary Information for “Simultaneous rift-scale inflation of a deep crustal sill network in Afar, East Africa”

A. La Rosa[1*], C. Pagli[1], H. Wang[2*], F. Sigmundsson[3] , V. Pinel[4], D. Keir[5,6*]

[1] Dipartimento di Scienze della Terra, Università di Pisa; Pisa, 56126, Italy
[2] College of Natural Resources and Environment, South China Agricultural University, Guangzhou, China
[3] Nordic Volcanological Center, Institute of Earth Sciences, University of Iceland; Reykjavik, Iceland
[4] University Grenoble Alpes, University Savoie Mont Blanc, CNRS, IRD, University Gustave Eiffel, ISTerre, Grenoble, 38000, France
[5] Dipartimento di Scienze della Terra, Università degli Studi di Firenze; Florence, 50121,Italy
[6] School of Ocean and Earth Science, University of Southampton, Southampton, UK

Corresponding Authors: Alessandro La Rosa (alessandro.larosa@dst.unipi.it**),** Hua Wang (ehwang@163.com**),** Derek Keir (D.Keir@soton.ac.uk**)**

Table of contents

The following supplementary material contains additional tests, figures and tables supporting our study. In particular, we show details on the spatial and temporal coverage of our InSAR dataset (Supplementary Figs. 1 and 3), along with raw and filtered time-series using different temporal filters. We show examples for time-series extracted from deforming areas (Supplementary Fig. 2), but also from areas affected by noise (Supplementary Fig. 5). We provide a supplementary method section describing the time-series cross-correlation analysis, along with the related results (Supplementary Fig. 4). We also provide the full 3D velocity maps and related uncertainties from inversion of InSAR and GNSS data and show three tests for different mesh design or GNSS datasets (Supplementary Figs. 6-11). As a further test we also show a simple inversion of InSAR velocity maps to obtain the EW and vertical components (Supplementary Fig. 12). We then provide details of the InSAR modeling, showing subsampled velocity maps used in the inversion, results for weighted and unweighted inversions, as also error calculations for each parameter of the four sills that we modeled (Supplementary Figs. 13-17 and Supplementary Table 1). Finally, we show results of alternative models based on magma buoyancy or a combination of buoyancy and sills inflation (Supplementary Figs. 18 and 19). Details on the seismicity from local and global catalogs are provided in Supplementary Fig. 20).

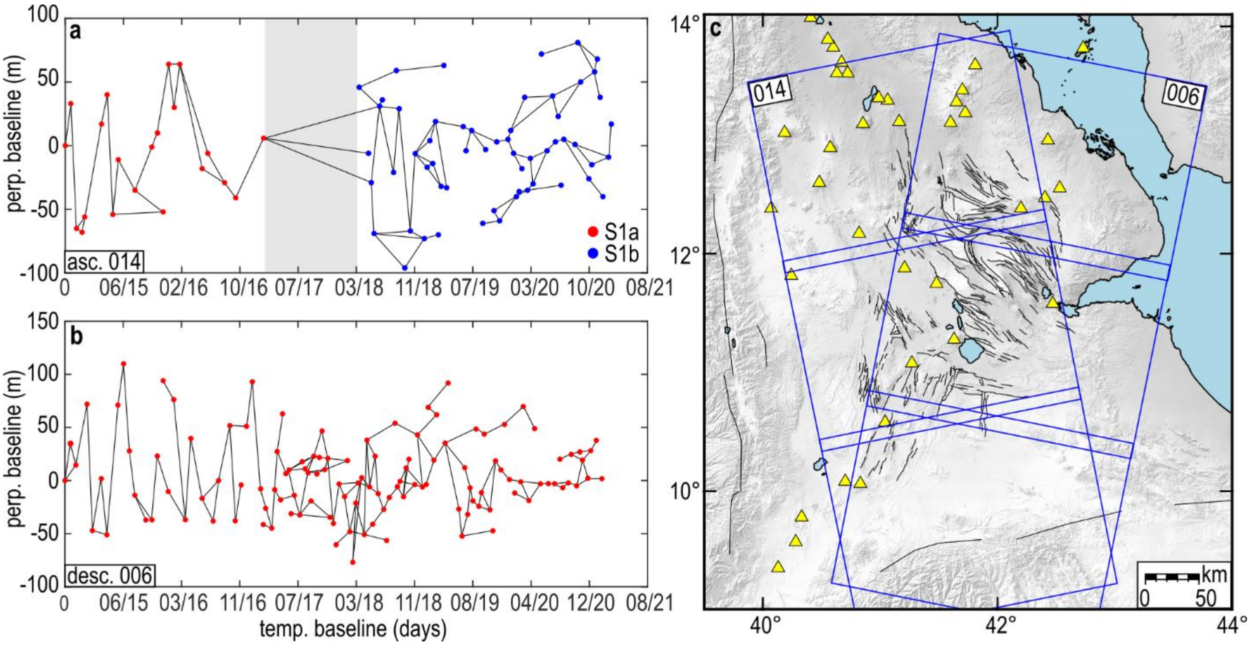

Supplementary Fig. 1. InSAR dataset. **a, b**. Interferograms networks for ascending and descending orbits. The y axes report the difference in the elevation of the satellite orbit during two adjacent acquisitions (perpendicular baseline). The x axis reports the date of the acquisitions and thus the temporal separation between them (temporal baselines). The gray area in **a** is the temporal gap in acquisitions during 2017. **c**, Orbits footprints in map view (blue polygons). Each rectangle represents a SAR frame covering a ~250 km-wide area. For our processing we stitched three frames for each track. The black lines are major faults and the yellow triangles are the quaternary volcanoes[21]. Topography is from the 1 arc-sec SRTM DEM[25].

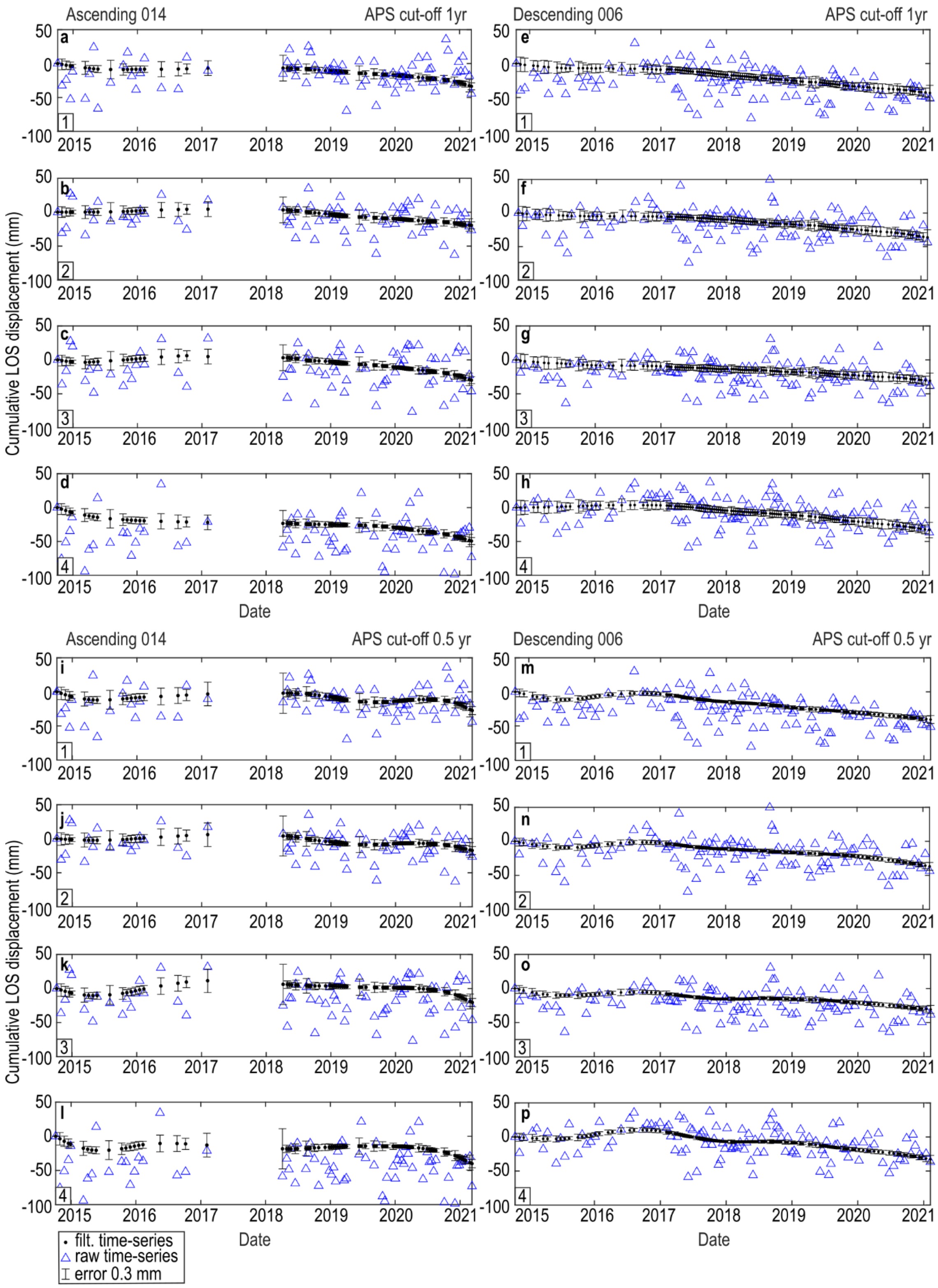

Supplementary Fig. 2. Time-series of cumulative LOS displacement**. a-h,** Atmospheric Phase Screen (APS) filter cut-off window of 1 year. **i-p,** APS filter cutoff window of 0.5 year**.** Time-series are numbered as in Fig. 1. The black dots are the filtered (filt.) time-series while the blue triangles are the raw time-series. Negative LOS displacements (range decrease) represent ground motions toward the satellite Errors (err., 2σ) are also reported as black bars. Filtered time-series with APS cut-off of 1 year**,** and associated uncertainties are provided in Supplementary Data 2-5. Pixels have the following coordinates: 1 = N12.1500°, E41.8835°; 2 = N11.7975°, E42.1330°; 3 = N11.6136°, E42.3807°; 4 = N11.9940°, E42.2748°.

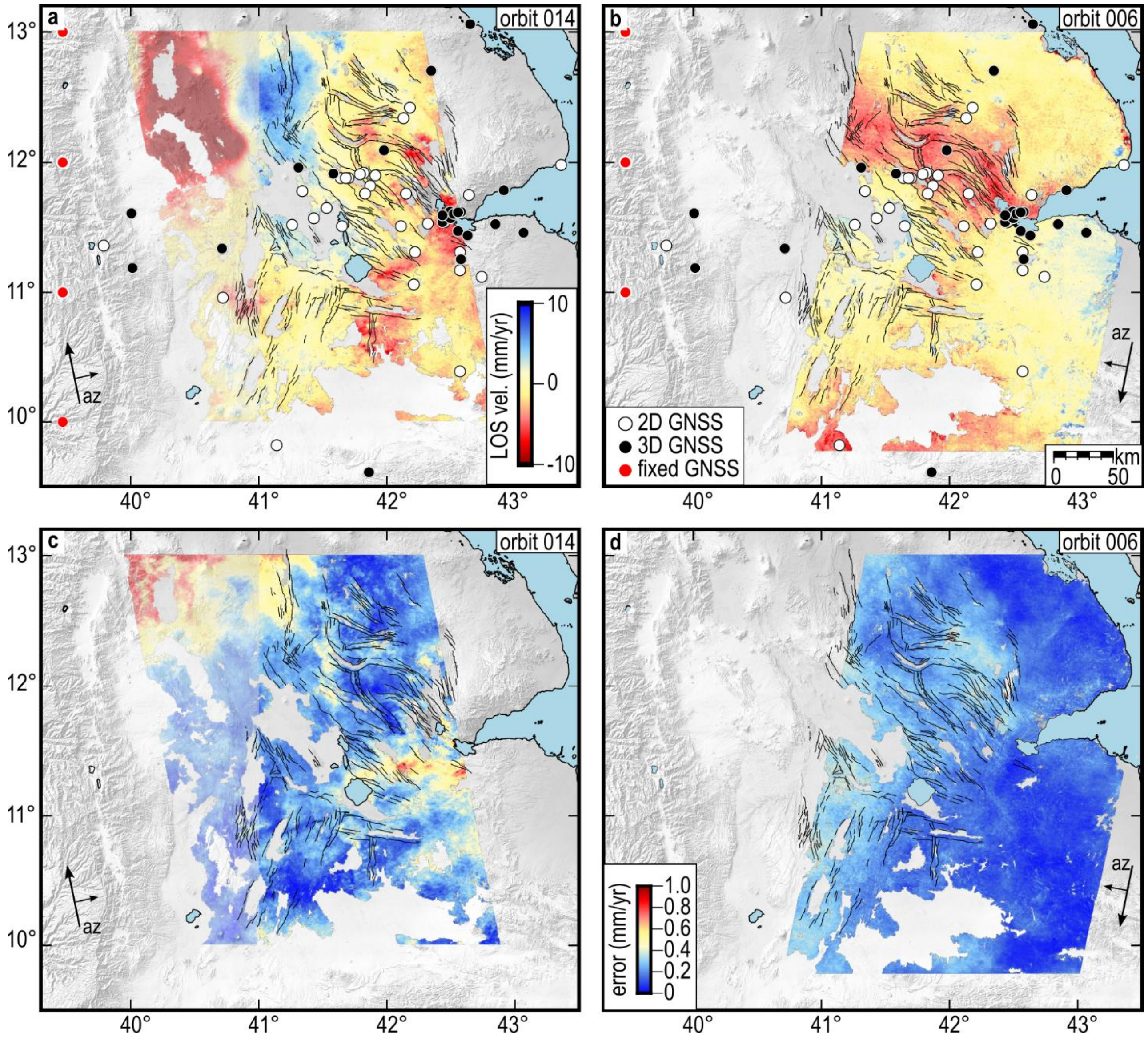

Supplementary Fig. 3. Geodetic dataset used in this study. **a, b,** Average LOS velocity (LOS vel.) from ascending and descending orbits, respectively, and GNSS data used for the 3D velocity calculations. **c, d,** standard deviation (σ) associated with the average LOS velocity maps. Negative values (range decrease) in the LOS data represent ground motions toward the satellite. The shaded area is that covering the DMH segment that we excluded from the 3D velocity field inversion. Black lines are major faults. The black arrows indicate the satellite geometry with the azimuth (az) direction. Topography is from the 1 arc-sec SRTM DEM[25]. The dataset is provided as Supplementary Data 5 and 6.

Supplementary Method

Time-series cross-correlation analysis

We calculated the Pearson's correlation coefficient on a moving window, between pairs of time-series (Supplementary Fig. 4). For this analysis, we used just the descending time-series as a large temporal gap in ascending of ~ 1 year (2017-2018, Fig. 1d) can provide false correlations[25]. Time-series were interpolated to maintain a constant temporal increment of 12 days, which corresponds to our minimum temporal separation between epochs. The Pearson's correlation coefficient (r) and the related statistical significance (t) were then calculated using a window size of 150 days (~5 months) as this ensures to investigate the magmatic sources of deformation and exclude any short-term contribution related to faulting[25]. The r value is representative of the strength of linear correlation between two variables and ranges between -1 and 1, with end-members values indicating strong anti-correlation and correlation, while zero indicates uncorrelated variables. For each r value we evaluated the statistical significance (t) through a t-test and considered values larger than a standard 0.05 as indicating r being highly significant, while values > 0.05 indicating r being not significant[26]. We then identified the onset of uplift in our time-series by searching for the first occurrence of r > 0.95 in each pair of time-series. Finally, we calculated correlation coefficient between time-series at one of the uplift sites (Allol) and one from the sites located just outside our interpreted uplift signal, reported in Supplementary Fig. 5. This test shows no clear evidence of correlation between the time-series, suggesting that the areas outside the uplift signal are dominated by noise.

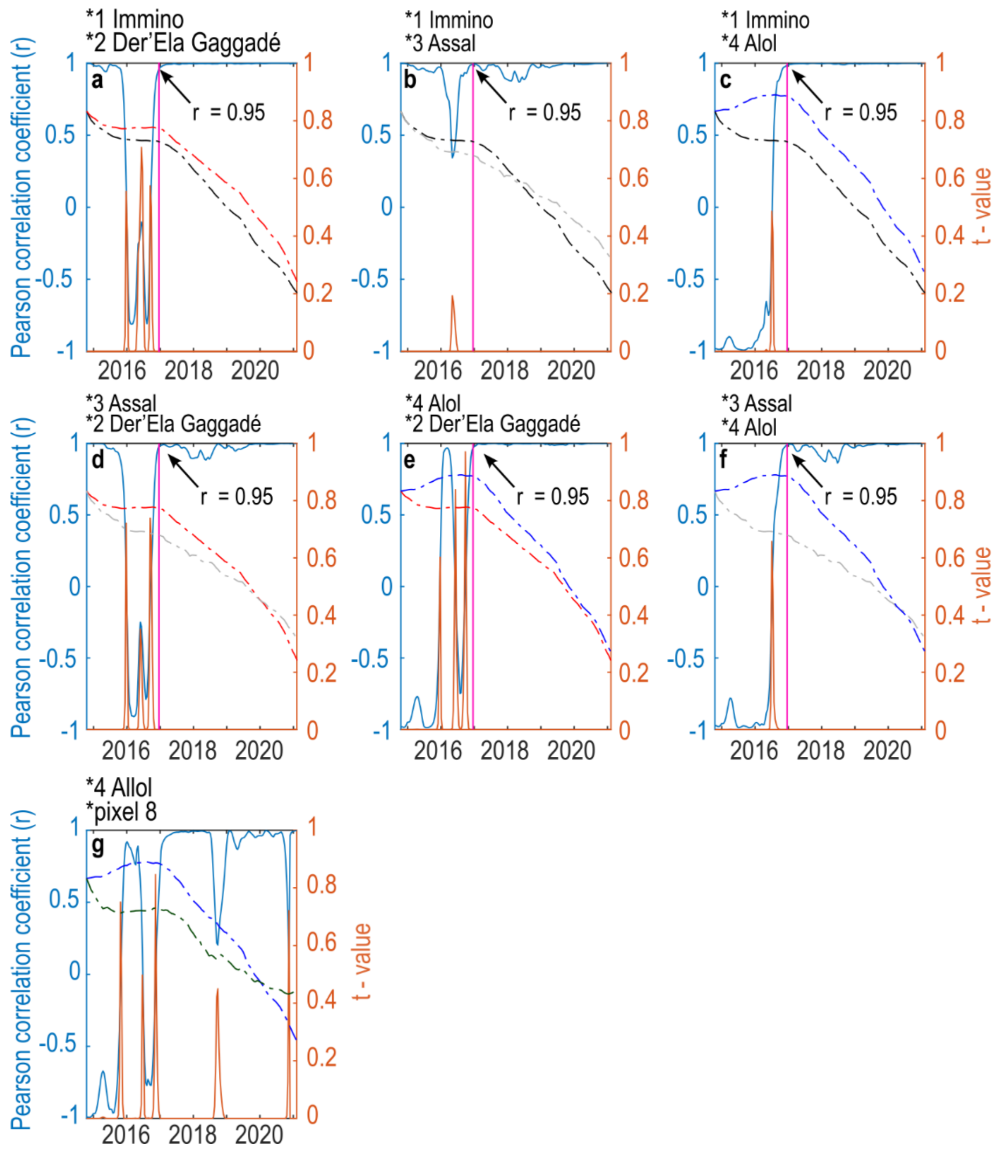

Supplementary Fig. 4. Time-series cross-correlation analysis. Analysis of correlation between pairs of descending time-series in order to identify similarities in the temporal pattern of deformation at the four uplifting areas in CA and to detect the eventual simultaneous onset of deformation[25]. The cyan y axis (left) and lines show the Pearson's correlation coefficient (r), while the orange y axis (right) and lines show the related statistical significance (t). The time-series (dashed lines) are scaled with the same y axis (not reported here) and color as in Fig. 1d. The magenta vertical lines mark the first occurrence of r = 0.95 in all the pairs of time-series, in December 2016. t values < 0.05 indicate statistically significant correlations during 2014 2015 and after December 2016, while they increase during December 2015-December 2016 likely due to higher local fluctuation and data variance for epochs of low deformation. The location of pixel 8 is shown in Supplementary Fig. 5.

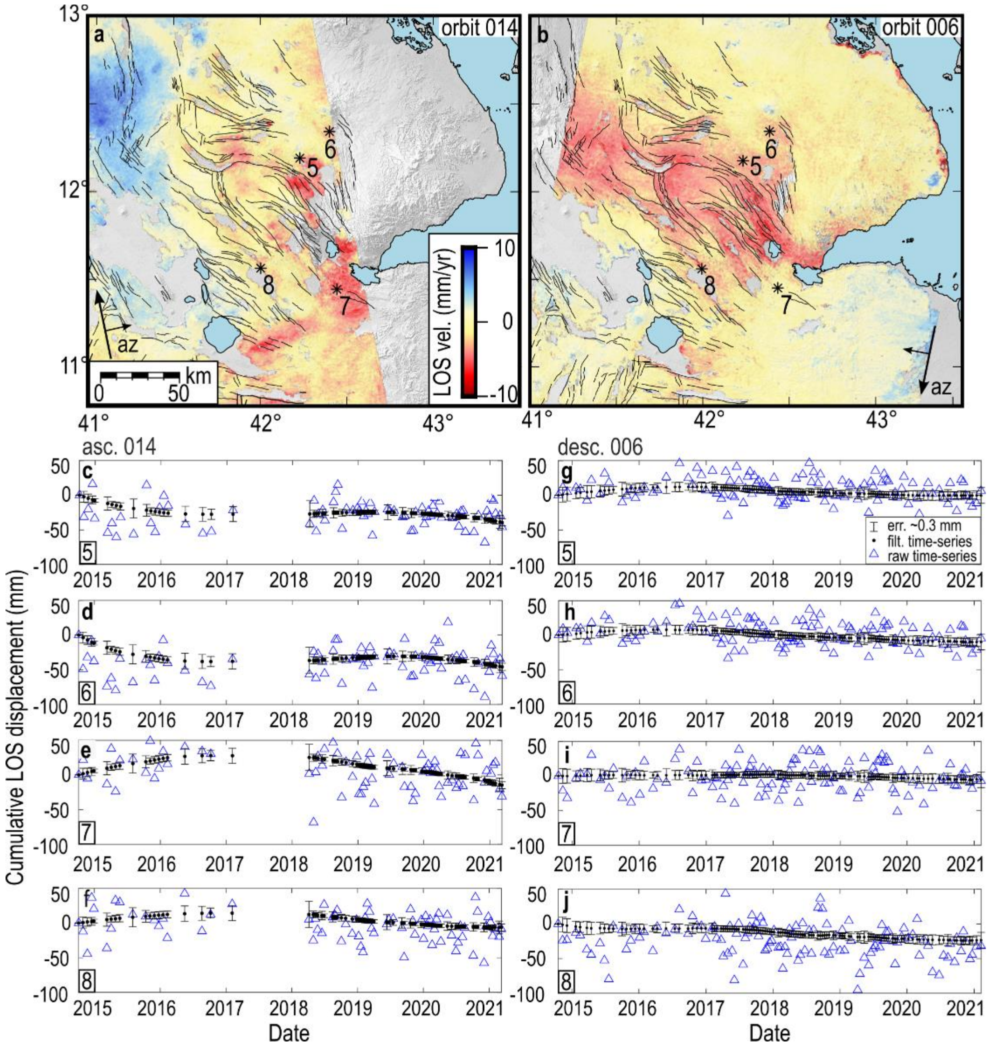

Supplementary Fig. 5 Time-series for areas located just outside the uplift signal. **a, b** Average LOS velocity (LOS vel.) map from ascending and descending orbits, as in Fig.1. The black arrows indicate the satellite geometry with the azimuth (az) direction. Topography is from the 1 arc-sec SRTM DEM[25]. **c-f** Time-series of cumulative LOS displacement from ascending orbit. **g-j** Time-series of cumulative LOS displacement from descending orbit. The black dots are the filtered (filt.) time series with an APS cut-off window of 1 year while the blue triangles are the raw time series. Errors (err., 2σ) are also reported as black bars. Negative LOS displacements (range decrease) represent ground motions toward the satellite. Filtered time-series with APS cut-off of 1 year**,** and associated uncertainties are provided in Supplementary Data 2-5. Pixels have the following coordinates: 5 = N12.3661°, E42.3652°; 6 = N12.3398°, E42.3827°; 7 = N11.4414°, E42.4251°; 8 = N11.5805°, E42.0069°. Ascending and descending time-series of points 5-8 show different trends, mainly fluctuating around zero motion and anyway showing trends inconsistent with the temporal pattern of the CA uplift.

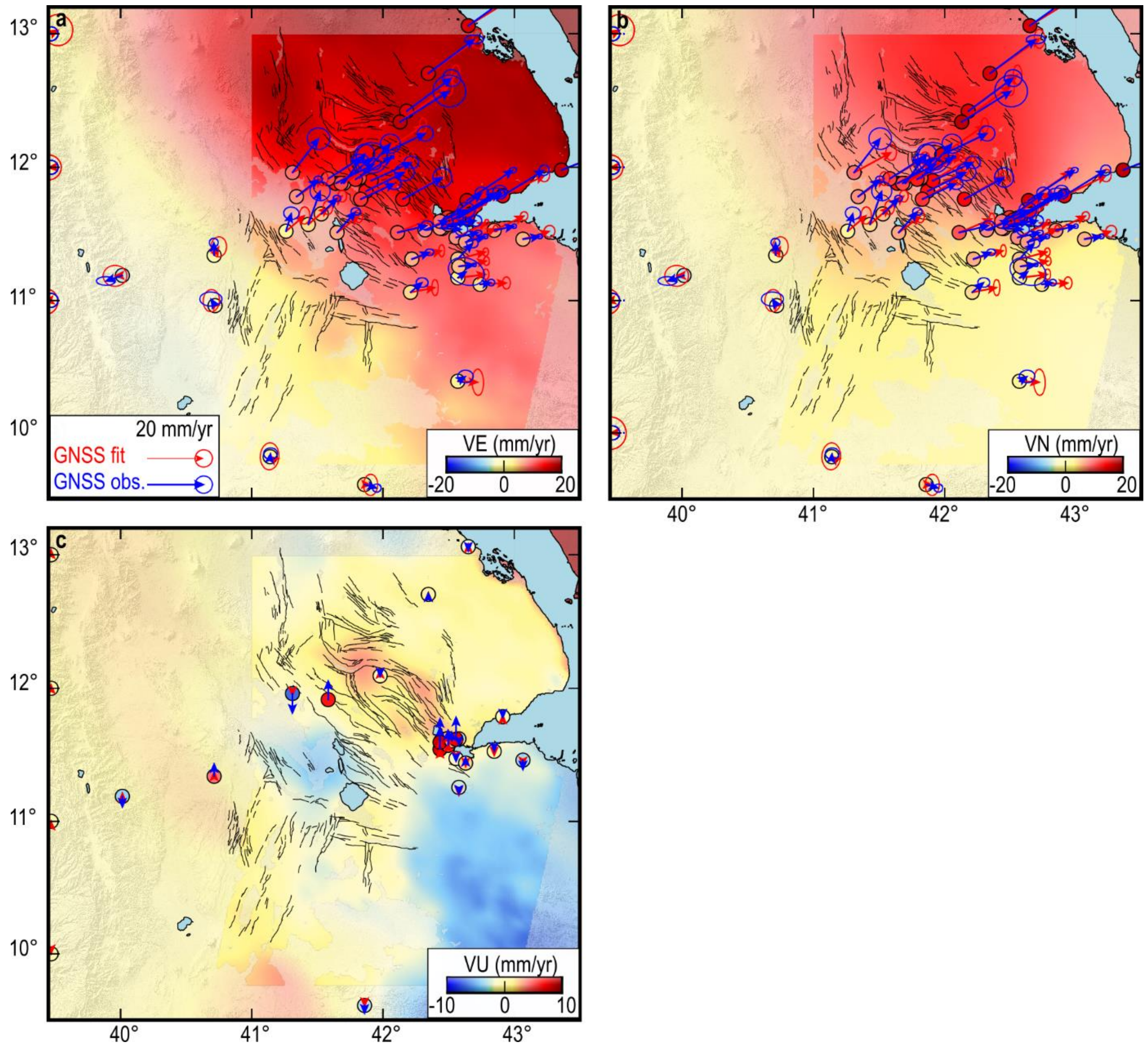

Supplementary Fig. 6. 3D velocity field inverted using a triangular mesh with spacing of 3 km. **a,** Eastward velocity (VE). **b,** Northward velocity (NV). **c,** Vertical velocity (VU, Up positive). The maps highlight the areas covered by at least one orbit, while shaded areas have no InSAR data points. Observed (obs.) and modeled GNSS velocities are reported as blue and red vectors, respectively, with related 95% confidence intervals. The colored dots are the measured GNSS velocity as represented by the colorbar. Note that vertical velocities are represented with a different colorscale. Black lines are major faults. Topography is from the 1 arc-sec SRTM DEM[25].

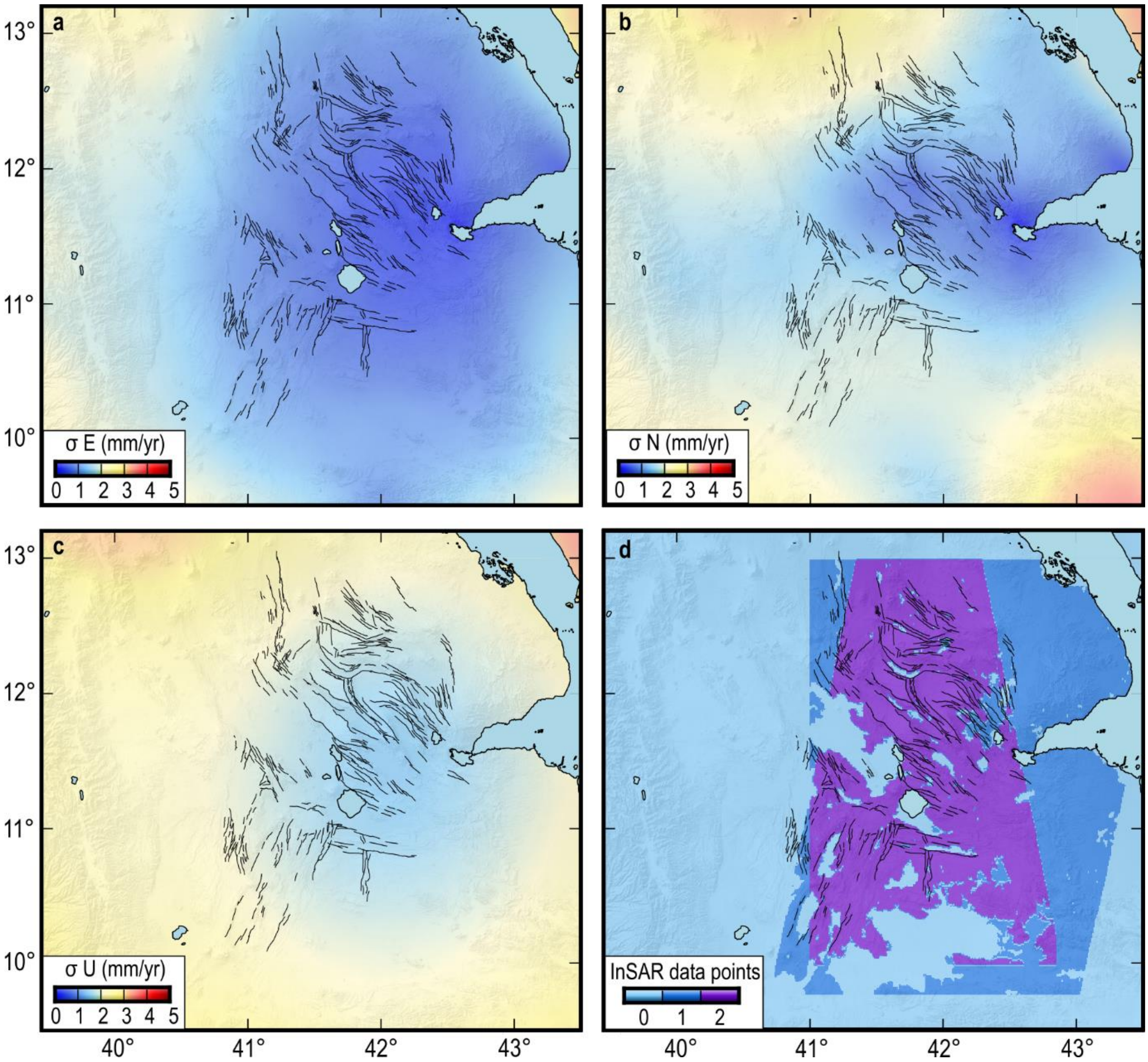

Supplementary Fig. 7. Uncertainties associated with the 3D velocity showed in Supplementary Fig. 6. **a,** standard deviation associated with the east (σE) velocity component. **b,** standard deviation associated with the north (σN) velocity component. **c,** standard deviation associated with the vertical (σU) velocity component. **d,** Number of InSAR data points, reported as number of InSAR velocity maps having a certain pixel. In CA where there is a better InSAR coverage, the uncertainties are lower. The vertical velocities in CA have $\sigma < 2$ mm/yr. Topography from the 1 arc-sec SRTM DEM[25].

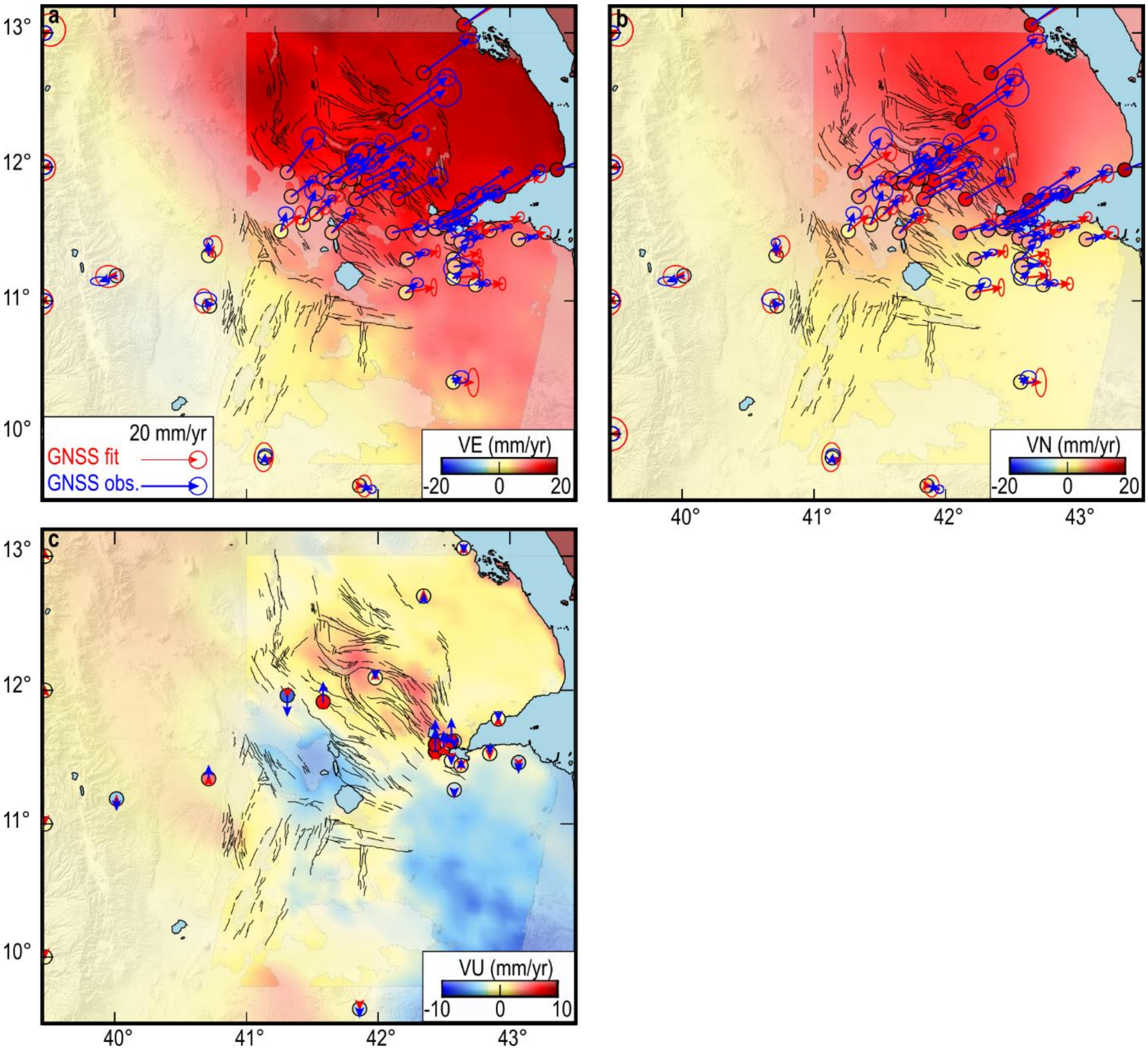

Supplementary Fig. 8. 3D velocity field inverted using a triangular mesh with spacing of 5 km. **a,** Eastward velocity (VE). **b,** Northward velocity (VN). **c,** Vertical velocity (VU, Up positive). The maps highlight the areas covered by at least one orbit, while shaded areas have no InSAR data points. Observed and modeled GNSS velocities are reported as blue and red vectors, respectively, with related 95% confidence intervals. The colored dots are the measured GNSS velocity as represented by the colorbar. Note that vertical velocities are represented with a different colorscale. Black lines are major faults. Topography is from the 1 arc-sec SRTM DEM[25].

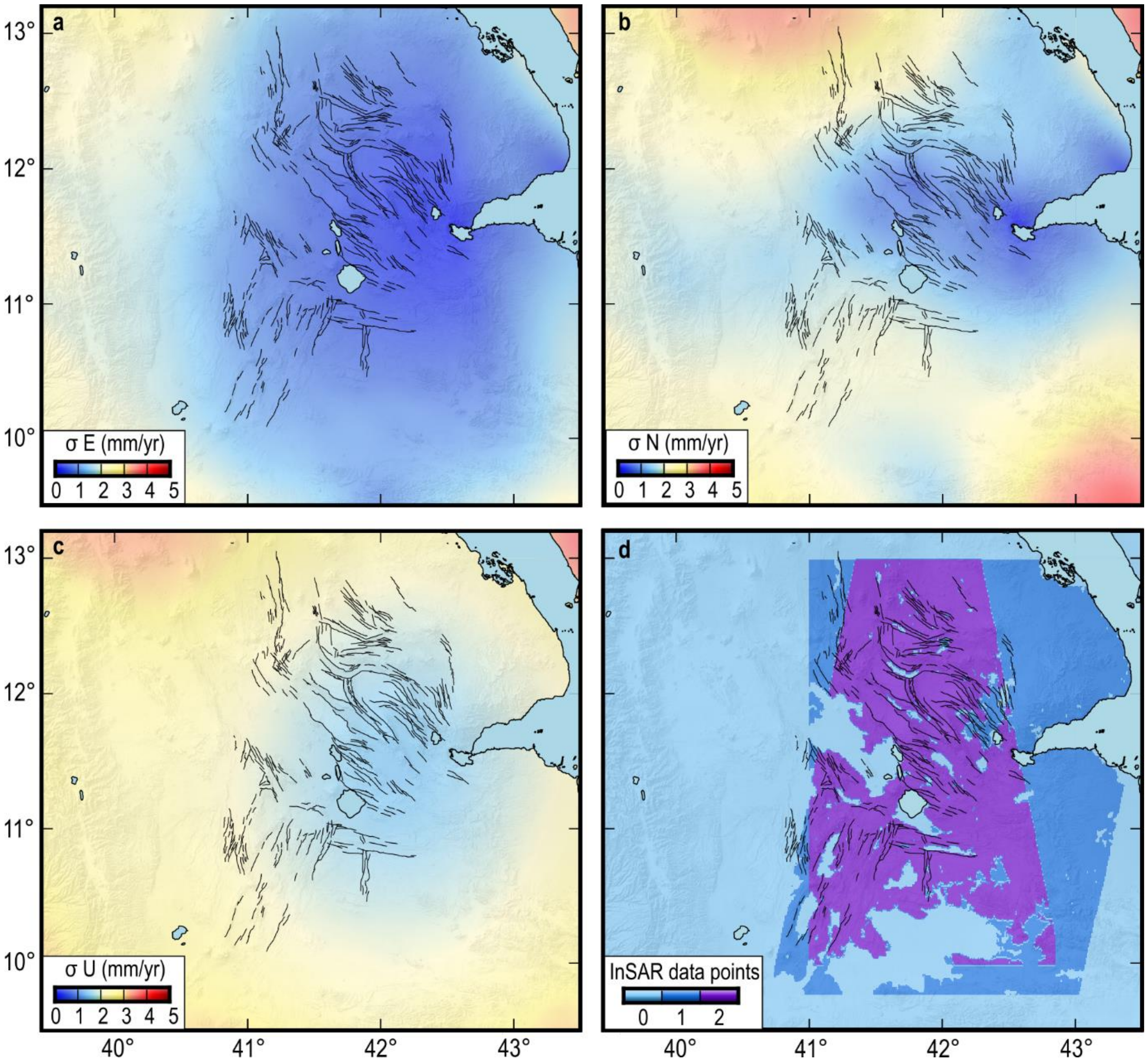

Supplementary Fig. 9. Uncertainties associated with the 3D velocity showed in Supplementary Fig. 8. **a,** standard deviation associated with the east (σE) velocity component. **b,** standard deviation associated with the north (σN) velocity component. **c,** standard deviation associated with the vertical (σU) velocity component. **d,** Number of InSAR data points, reported as number of InSAR velocity maps having a certain pixel. In CA where there is a better InSAR coverage, the uncertainties are lower. The vertical velocities in CA have σ < 2 mm/yr. Topography is from the 1 arc-sec SRTM DEM[25].

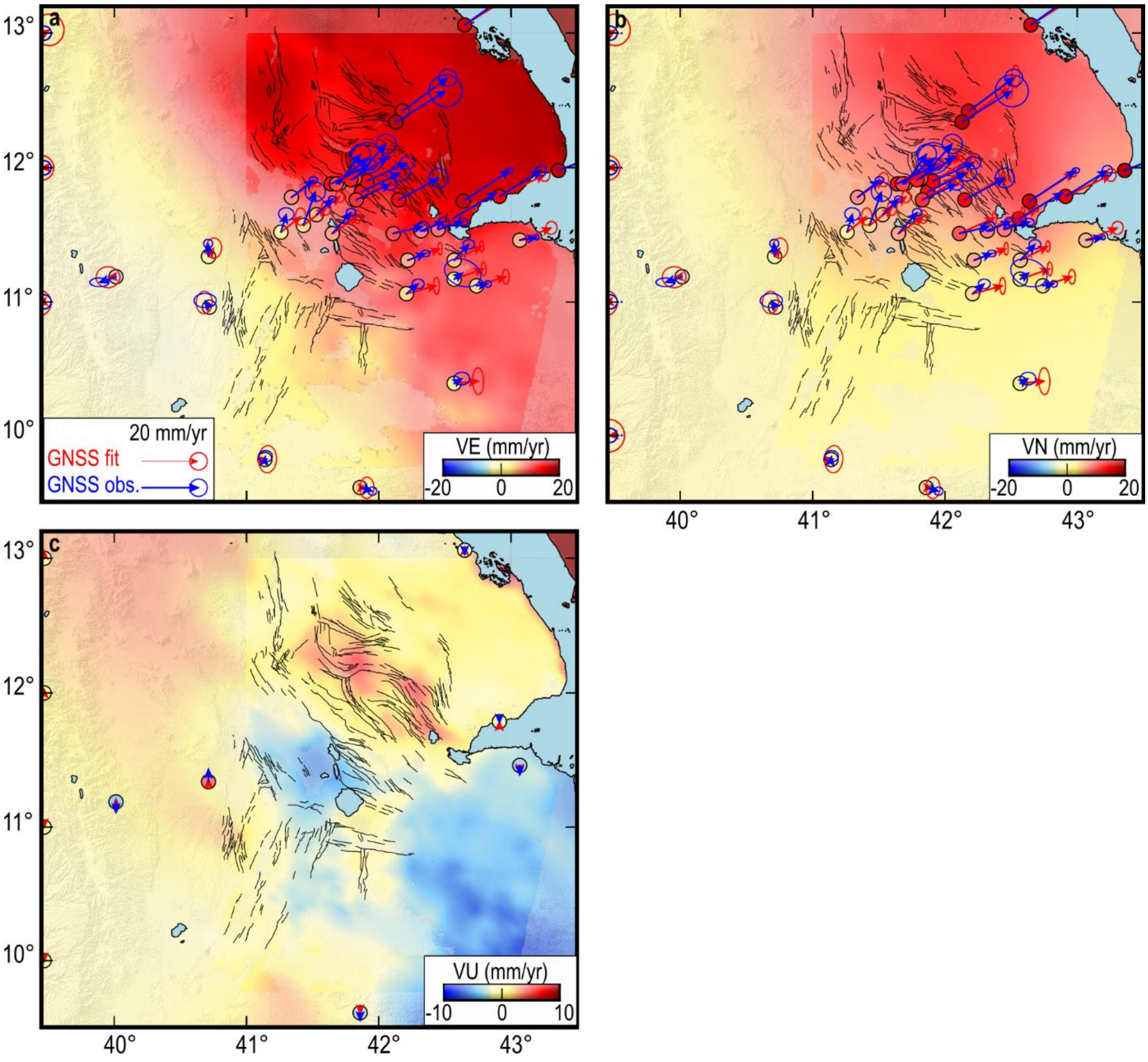

Supplementary Fig. 10. 3D velocity field calculated with GNSS vertical velocities only at the margins of the study area and not on the anomaly in Central Afar (panel c shows the GNSS sites that include the vertical velocity). **a,** Eastward velocity (VE). **b,** Northward velocity (VN). **c,** Vertical velocity (VU, Up positive). Bright areas in the maps highlight the areas covered by at least one InSAR orbit, while shaded areas have no InSAR data points. Observed and modeled GNSS velocities are shown as vectors with 95% confidence intervals. The colored dots at the base of each vector are the observed GNSS velocities scaled by the colorbar. Note that in c, vertical velocities are shown using a different color scale. Black lines are major faults. Topography is from the 1 arc-sec SRTM DEM[25].

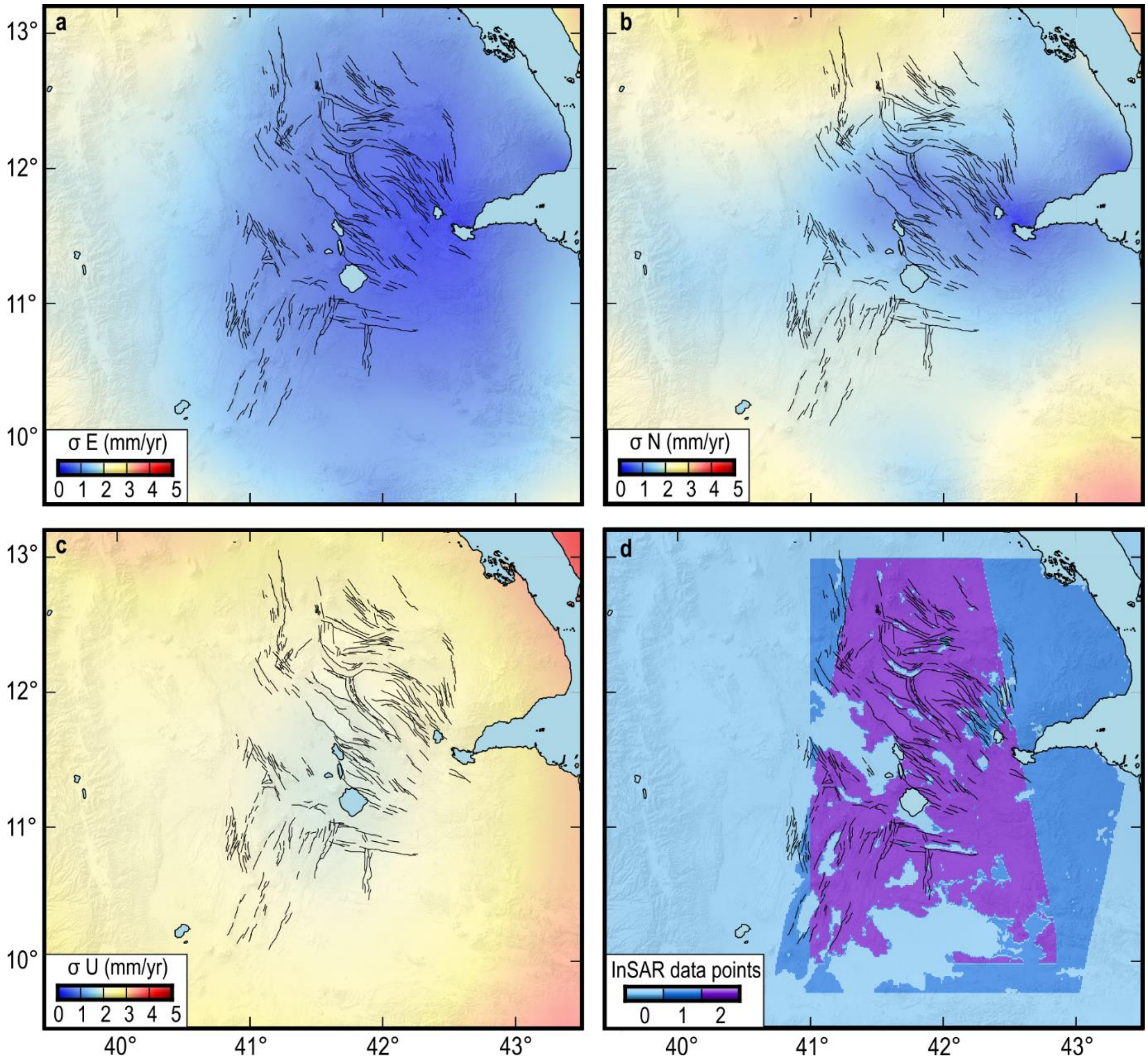

Supplementary Fig. 11. Uncertainties associated with the 3D velocity showed in Supplementary Fig. 10. **a,** standard deviation associated with the east (σE) velocity component. **b,** standard deviation associated with the north (σN) velocity component. **c,** standard deviation associated with the vertical (σU) velocity component. **d,** Number of InSAR data points, reported as number of InSAR velocity maps having a certain pixel. In CA where there is a better InSAR coverage, the uncertainties are lower. In this test, the σ values of the vertical velocities slightly increase in CA but remain < 3 mm/yr. Topography is from the 1 arc-sec SRTM DEM[25].

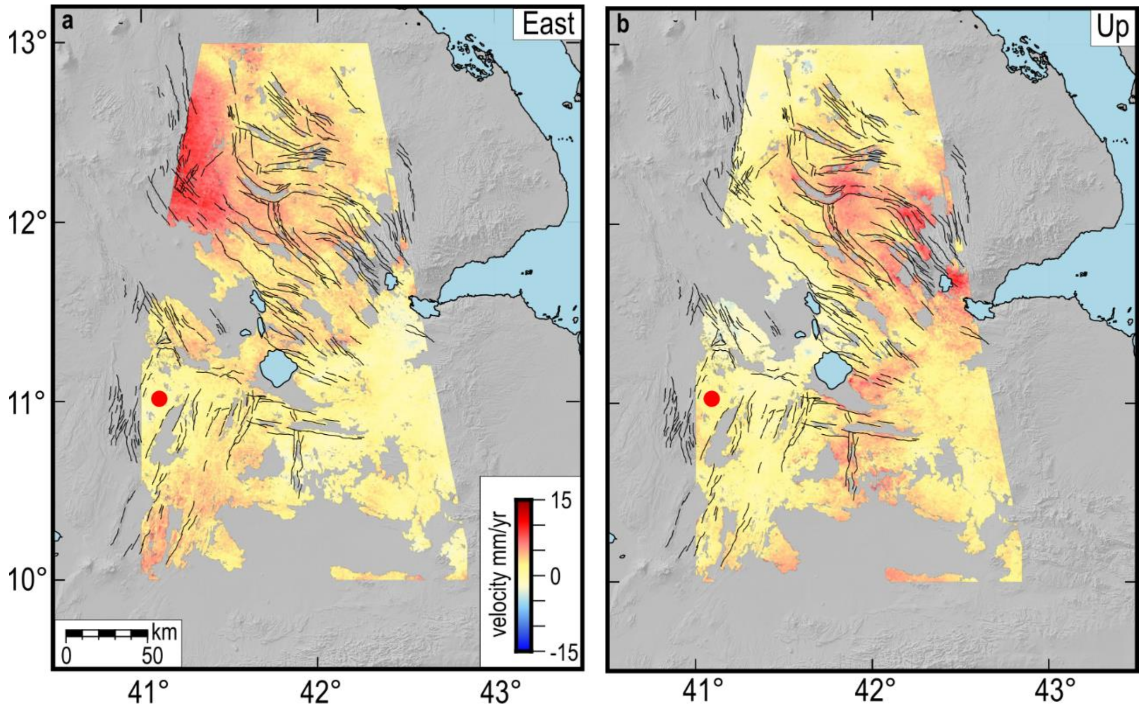

Supplementary Fig. 12. East and Vertical velocity maps obtained by inversion of ascending and descending InSAR LOS velocity maps and assuming no North-South component. Velocities are calculated just for pixels which are common to both maps. The velocities are not relative to fixed Nubia and the reference point is marked by the red dot. Topography is from the 1 arc-sec SRTM DEM[25].

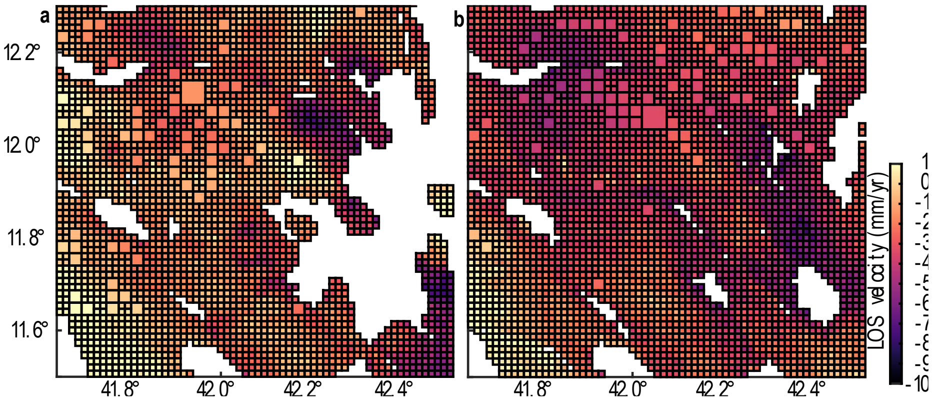

Supplementary Fig. 13. Average LOS velocity maps subsampled using a quadtree partitioning algorithm and used as input for the inversion. **a,** Ascending orbit 014. **b,** Descending orbit 006. Negative values (range decrease) in the LOS data represent a reduction of the distance between satellite and Earth's surface and thus ground motions toward the satellite.

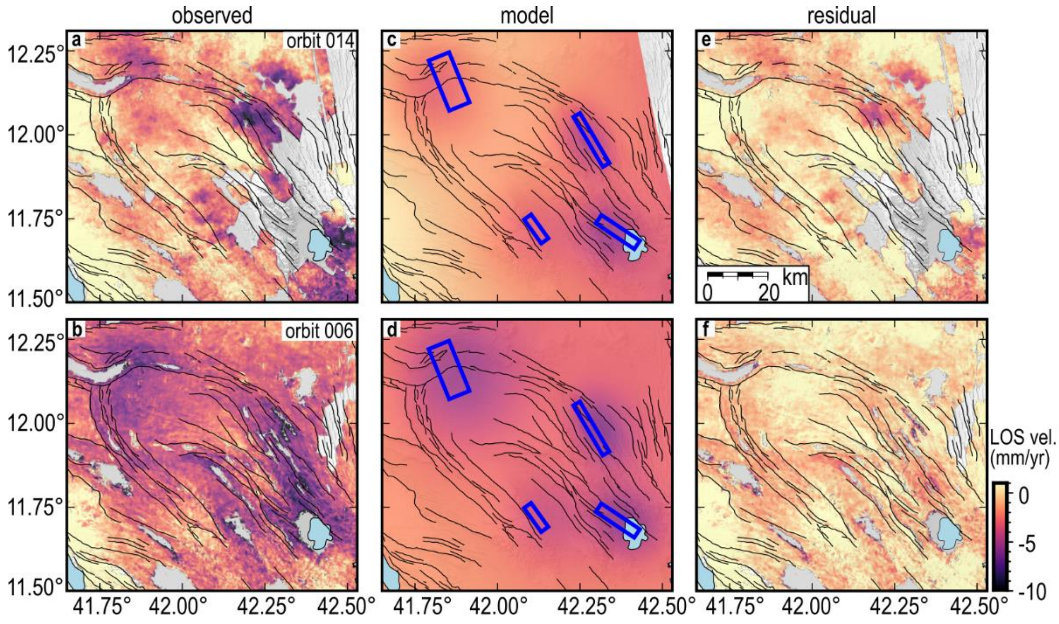

Supplementary Fig. 14. Geodetic modeling result (Model 1). **a, b,** Observed InSAR deformation from ascending (014) and descending (006) orbits, respectively. **c, d,** best fit models. **e, f,** Residuals. Black lines are major faults while the blue polygons are the surface projections of the four best-fit sills. Negative values (range decrease) in the LOS data represent a reduction of the distance between satellite and Earth's surface and thus ground motions toward the satellite. Topography is from the 1 arc-sec SRTM DEM[25].

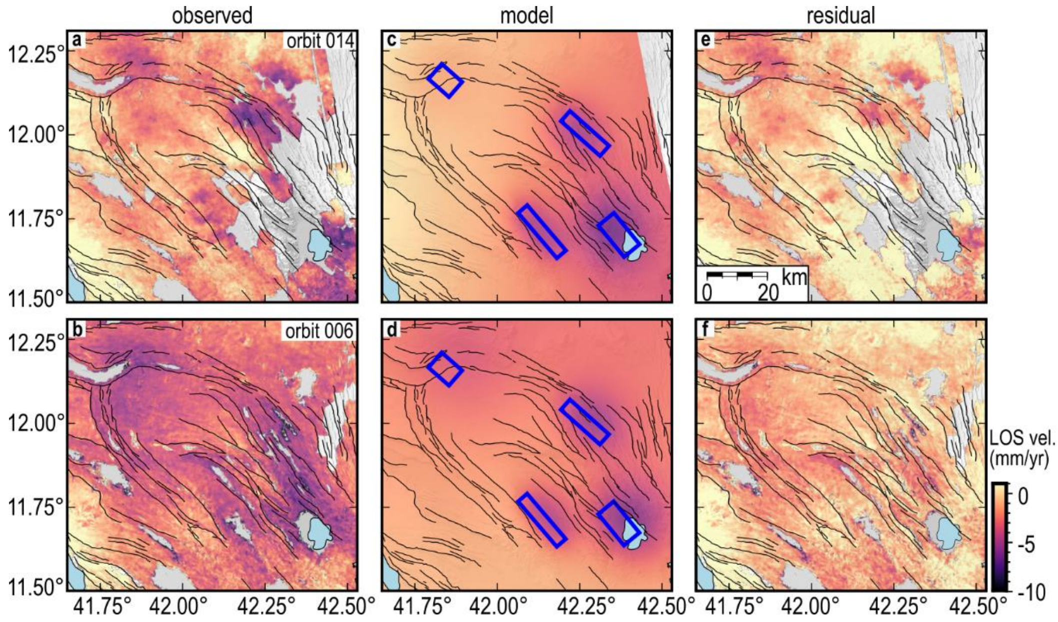

Supplementary Fig. 15. Geodetic modeling result (Model 2). **a, b,** Observed InSAR deformation from ascending (014) and descending (006) orbits, respectively. **c, d,** best fit models. **e, f,** Residuals. Black lines are major faults while the blue polygons are the surface projections of the four best-fit sills. Negative values (range decrease) in the LOS data represent a reduction of the distance between satellite and Earth's surface and thus ground motions toward the satellite. Topography is from the 1 arc-sec SRTM DEM[25].

#	Lon. (°)	Lat. (°)	D (km)	L (km)	W (km)	Strike (°)	Dip (°)	op. (mm/yr)	vol. (m^3/yr)
**Model 1**									
**1**	41.8532	12.1592	29.72	17.84	7.32	N337.7°E	9NE	62.8	8.2×10^6
**2**	42.1170	11.7192	23.59	9.72	2.90	N324.1°E	2SW	91.0	2.6×10^6
**3**	42.3646	11.7101	14.71	15.28	3.34	N303.6°E	0.8SW	39.7	2.0×10^6
**4**	42.2844	11.9848	11.26	19.11	2.57	N329.3°E	10NE	31.5	1.5×10^6
					**rms 006 =** 1.05				
					**rms 014 =** 1.37				
**Model 2**									
**#**	**Lon. (°)**	**Lat. (°)**	**D(km)**	**L(km)**	**W(km)**	**Strike (°)**	**Dip (°)**	**op. (mm/yr)**	**vol. (m^3/yr)**
**1**	41.8420	12.1623	28.27	8.99	6.62	N311.4°E	4NE	44.3	2.6×10^6
**2**	42.1355	11.7109	9.04	19.67	3.98	N319.3°E	8NE	15.8	1.2×10^6
**3**	42.3689	11.7015	17.30	13.40	6.68	N320,4°E	8.3NE	41.7	3.7×10^6
**4**	42.2659	12.0041	12.16	17.27	4.48	N311.5°E	4.6NE	21.1	1.6×10^6
					**rms 006 =** 1.35				
					**rms 014 =** 1.39				

Supplementary Table 1. Best-fit parameters for the two models described in the Methods section. Lon. = longitude of the sill centroid, Lat. = latitude of the sill centroid, D = depth, L = length, W = width, Op = opening rate, vol = volume change rate. The Root Mean Square (RMS) misfits are in mm/yr.

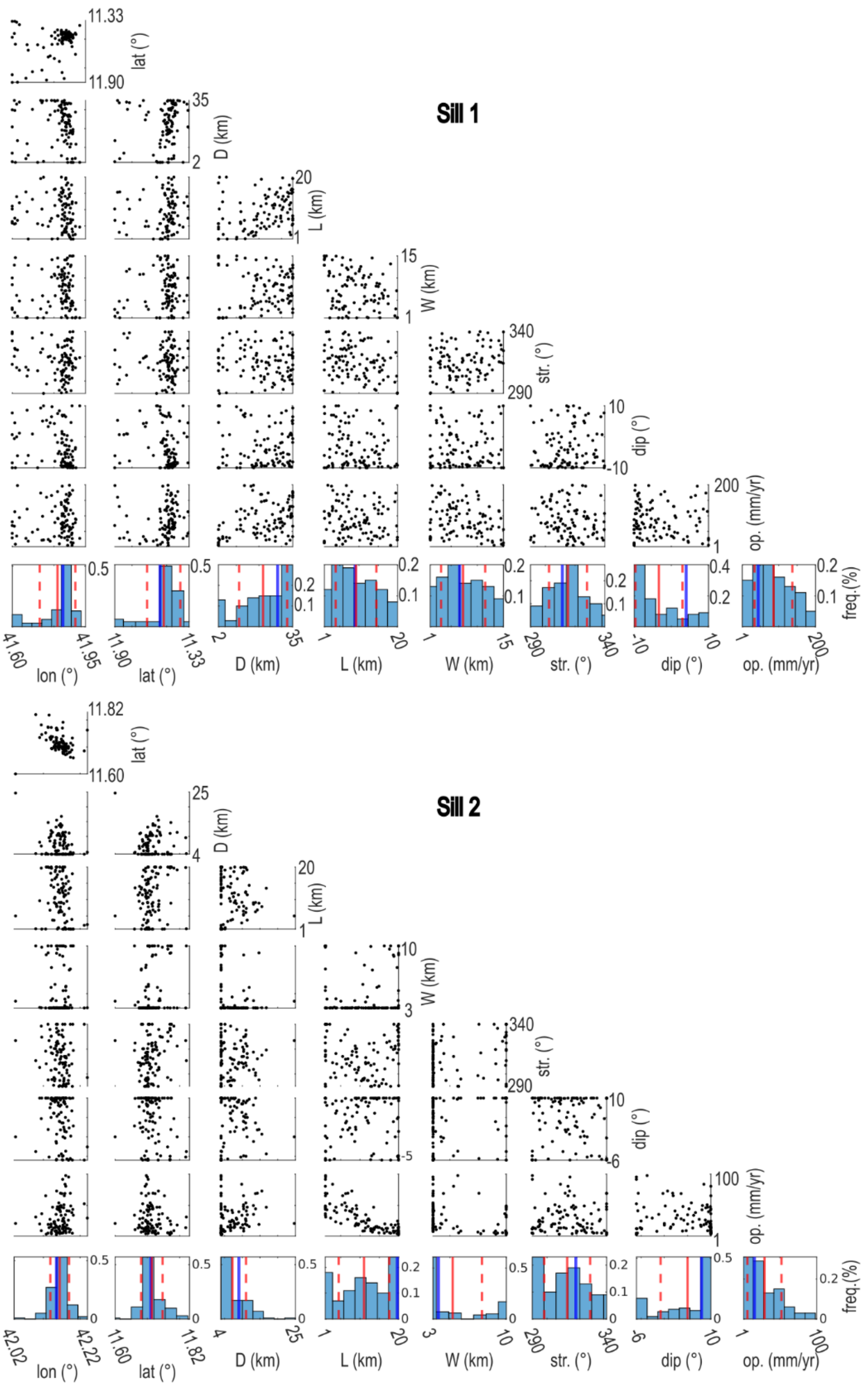

Supplementary Fig. 16. Distribution and frequency resulting from the uncertainty calculation for the parameters of sills 1 and 2**.** The blue solid bars are the best-fit parameter values as in Model 2, while the red dashed bars are the standard deviations (2σ) around the mean (red solid line). Axes limits correspond to the search bounds used for the error calculation. lon. = longitude of the sill centroid, lat. = latitude of the sill centroid, D = depth, L = length, W = width, str. = strike, op. = opening rate. Some model parameters have large σ and trade-offs between parameters are also present. Depth of sill 1 for example shows large σ and spread in the data distribution caused by trade-off with the opening rate parameter. However, the mean and standard deviation of the sill depths distribution is consistent with our best-fit model: mean depth and 2σ of 22±11 km for sill 1, 7±4 km for sill 2. The results of the error calculation and σ values are provided as Supplementary Data 8.

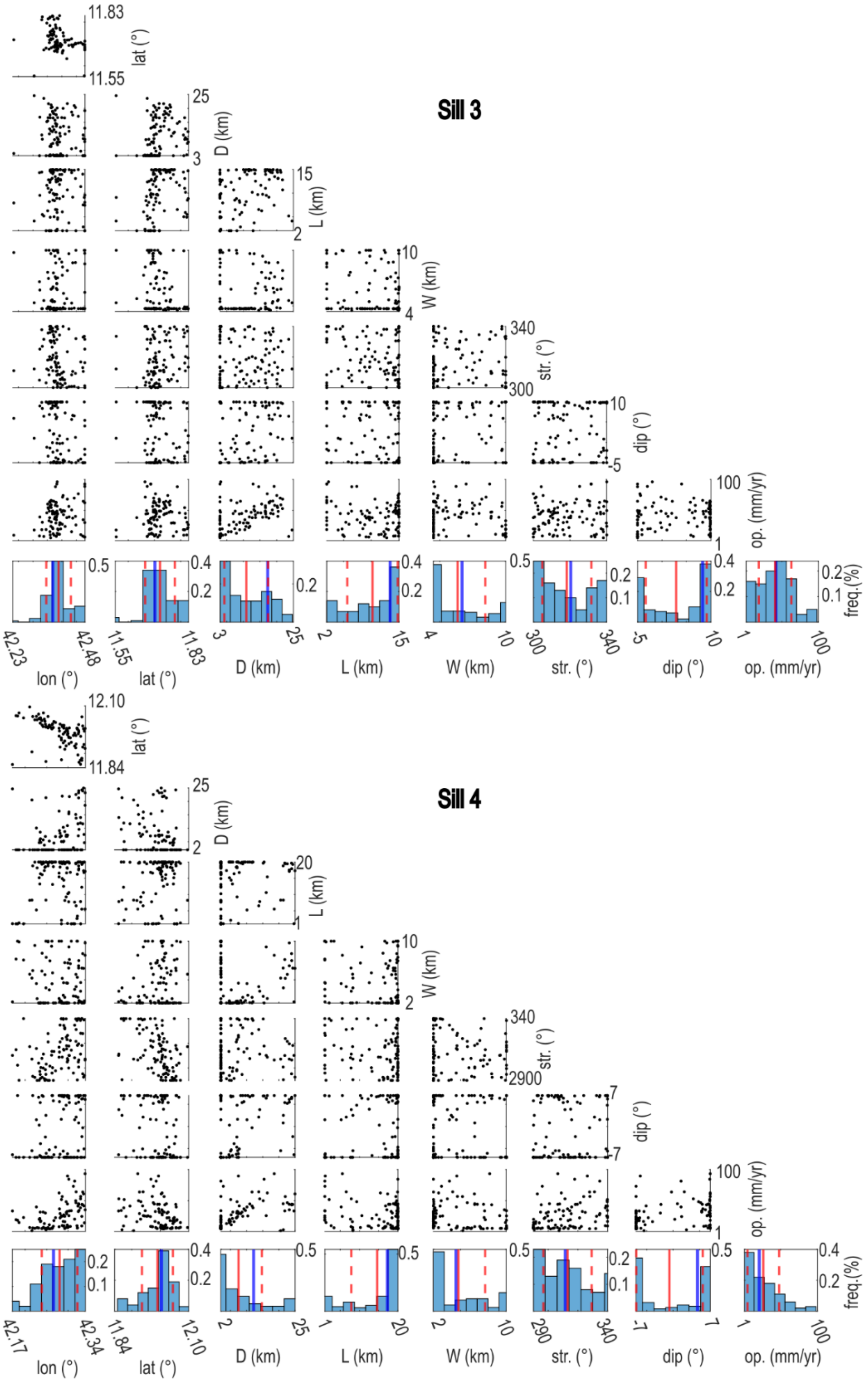

Supplementary Fig. 17. Distribution and frequency resulting from the uncertainty calculation for the parameters of sills 3 and 4**.** The blue solid bars are the best-fit parameter values as in Model 2, while the red dashed bars are the standard deviations (2σ) around the mean (red solid line). Some model parameters have large σ and trade-offs between parameters are also present. Axes limits correspond to the search bounds used for the error calculation. lon. = longitude of the sill centroid, lat. = latitude of the sill centroid, D = depth, L = length, W = width, str. = strike, op. = opening rate. Depths of sill 3 and 4 for example show large σ and spread in the data distribution caused by trade-off with the opening rate parameter. However, the mean and standard deviation of the sill depths distribution is consistent with our best-fit model: mean depth and 2σ of 11±6 for sill 3, and 7.5±7 km for sill 4. The results of the error calculation and σ values are provided as Supplementary Data 8.

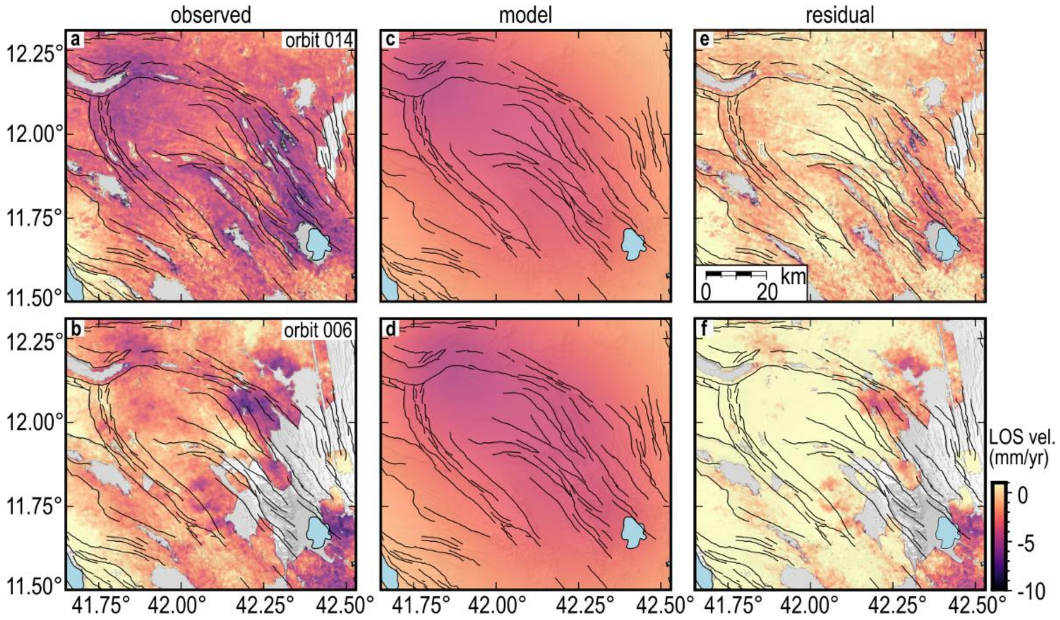

Supplementary Fig. 18. Finite Element Model of deformation of the buoyancy component induced by magma accumulation at the boundary between an elastic and an inviscid layer. **a, b** Observed InSAR deformation from ascending and descending orbits, respectively. **c, d** predicted flexure model deformation projected along the satellite LOS direction for the ascending (**a**) and descending (**b**) satellite orbits utilized in this study. **e, f** residuals. Topography is from the 1 arc-sec SRTM DEM[25].

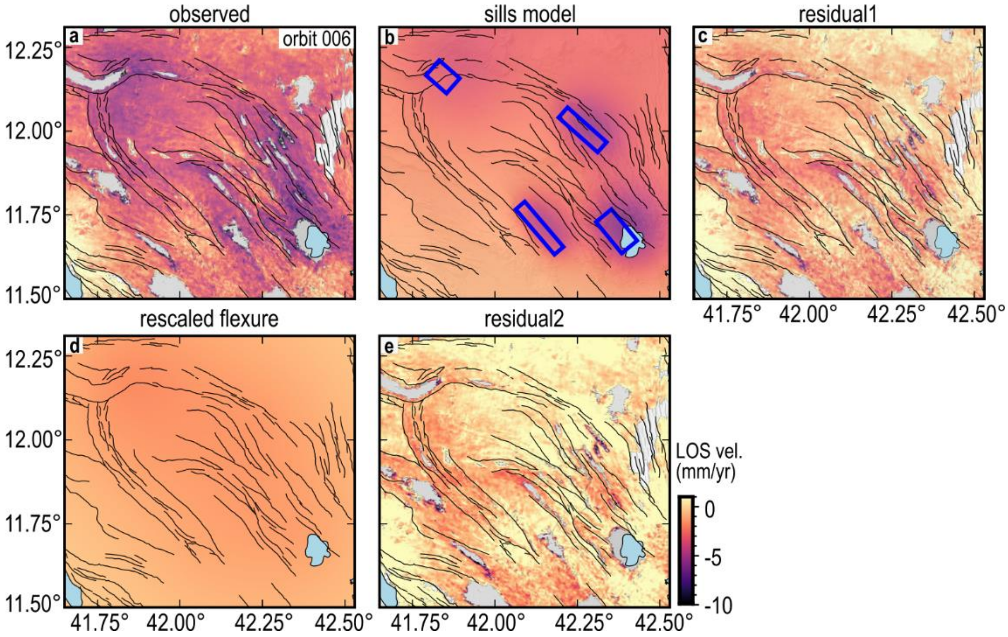

Supplementary Fig. 19. Test results of a combined sill inflation and buoyancy to fit the observation from descending orbit 006. **a-c,** InSAR observation model and residual 1 as in Fig. 1 and Supplementary Fig. 15. The blue polygons are the surface projection of the four best-fit Okada sources (sills). **d,** rescaled flexure model to fit the amplitude of long-wavelength residual signal in c). For the rescaling we multiplied the model showed in Supplementary Fig. 18 by a factor of 0.35. **e,** residual 2 obtained by subtracting the rescaled flexure from residual 1. The total RMS misfit for residual2 is 1.03 mm/yr. Topography is from the 1 arc-sec SRTM DEM[25].

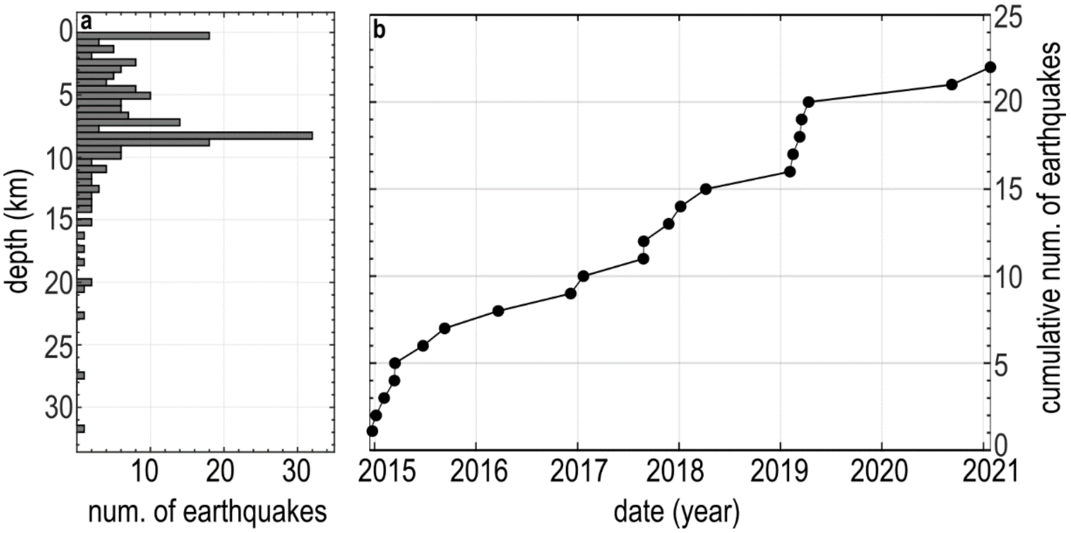

Supplementary Fig. 20. Seismicity distribution in Central Afar. **a,** Depth distribution of earthquakes recorded by a temporary seismic network and relocated by ref. 27. The events occurred in the area between latitudes N11°-N12.8° and longitudes E41°-E42.7°, during 2005-2010. The bin size of histograms is 500 m. **b,** Cumulative number of earthquakes recorded by global seismic networks during the same time-period of InSAR (2014-2021). The curve does not show particular increases in the seismic rate during the sills intrusion, after December 2016. These earthquakes are included in the catalog provided as Supplementary Data 1.
