# Supplementary material for: Simultaneous rift-scale inflation of a deep crustal sill network in Afar, East Africa
Source: Nat Commun. 2024 May 20;15:4287. doi: 10.1038/s41467-024-47136-4 (PMC11893136; doi:10.1038/s41467-024-47136-4)
Supplement: Supplementary file 3 — Description of Additional Supplementary Files [file 41467_2024_47136_MOESM3_ESM.pdf]

Description of Additional Supplementary Files

File Name: Supplementary Data 1
Description: Earthquakes catalog for Central Afar

File Name: Supplementary Data 2
Description: Time-series of cumulative LOS displacement from descending orbit 006

File Name: Supplementary Data 3
Description: Uncertainties associated with time-series from descending orbit 006

File Name: Supplementary Data 4
Description: Time-series of cumulative LOS displacement from ascending orbit 014

File Name: Supplementary Data 5
Description: Uncertainties associated with time-series from ascending orbit 014

File Name: Supplementary Data 6
Description: Average LOS velocities and related uncertainties from descending orbit 006

File Name: Supplementary Data 7
Description: Average LOS velocities and related uncertainties from ascending orbit 014

File Name: Supplementary Data 8
Description: Parameters distribution of 100 models resulting from the uncertainty calculation for latitude, longitude, depth, length, width, strike, dip angle and opening of the four sills
